# Intrinsic Plasticity-Driven Neuroadaptive Asymptotic Tracking Control for a Class of Uncertain Robotic Manipulators

**DOI:** 10.3390/s26144643

**Published:** 2026-07-22

**Authors:** Qing Chen, Xiangyang Tan, Shuaicheng Hou, Meiyi Qing, Zuojin Li

**Affiliations:** School of Electronic and Electrical Engineering, Chongqing University of Science and Technology, Chongqing 401331, China; 2021049@cqust.edu.cn (Q.C.); 2024204028@cqust.edu.cn (X.T.); shuaichenghou@cqust.edu.cn (S.H.); qingmeiyi323@126.com (M.Q.)

**Keywords:** echo state network, intrinsic plasticity, asymptotic tracking control, cycle reservoir, RISE

## Abstract

This paper proposes a novel neuroadaptive asymptotic tracking control method for a class of uncertain multi-input multi-output (MIMO) robotic manipulators. Firstly, an intrinsic plasticity (IP)-driven cycle echo state network (ESN) is constructed. The intrinsic plasticity mechanism can adaptively adjust neuronal excitability, enhancing the network’s ability to capture complex time-varying dynamics. Meanwhile, the cycle reservoir structure significantly reduces the number of neural connections, thus improving computational efficiency. Secondly, the proposed IP-driven cycle ESN is integrated with the robust integral of the sign of the error (RISE) framework to form a neuroadaptive controller. The IP-driven cycle ESN serves to dynamically approximate the unknown nonlinearities inherent in the robotic system, whereas the RISE term compensates for approximation errors and external disturbances to ensure satisfactory robust performance. Then, a rigorous stability analysis is given to demonstrate that the proposed controller can achieve asymptotic convergence of the tracking error. Finally, simulation experiments are conducted on two typical two-joint manipulators to evaluate the performance of the proposed method. Comparative results demonstrate that, in contrast to the Radial Basis Function Neural Network (RBFNN)-based PI control method, the proposed method achieves faster error convergence rate, higher tracking precision, and smoother control inputs. The results highlight the effectiveness of combining the approximation capability of the IP-driven cycle ESN with the robust compensation capability of the RISE framework for high-precision control of uncertain nonlinear robotic systems.

## 1. Introduction

With the rapid advancement of industrial automation, intelligent manufacturing, medical rehabilitation, and service robotics, robotic systems have become deeply integrated into modern production and daily life [1]. However, in practical applications, these systems are inevitably subjected to complex uncertainties, including unmodeled dynamics, intrinsic parameter variations, and external time-varying disturbances. Traditional control strategies often struggle to maintain satisfactory control performance in the presence of these complex uncertainties. Consequently, achieving high-precision trajectory tracking under dynamic, uncertain conditions remains a critical challenge. To address this issue, a variety of advanced control strategies have been extensively investigated. For instance, semi-model-free adaptive backstepping dynamic sliding mode control was introduced to handle uncertain manipulator dynamics [2]. Similarly, the fixed-time control method has been deployed to mitigate lumped disturbances and actuator faults [3], while global integral terminal sliding mode approaches have been developed to guarantee finite-time convergence under unknown parameters [4]. Despite their success, the majority of existing schemes remain constrained by the requirement of known system structures or predefined bounding functions. To circumvent these limitations, neural network (NN)-based control methods have attracted increasing attention in recent years, particularly in the field of robot control. Owing to their powerful nonlinear approximation capability, NNs are uniquely suited to reconstruct unknown dynamics in complex robotic systems [5,6]. Meanwhile, their inherent self-learning adaptability provides an effective framework for capturing unmodeled dynamics and real-time parameter variations [7,8]. By leveraging these features, NN-based controllers exhibit remarkable robustness against severe coupling effects and external disturbances [9,10,11], making them a cornerstone of modern intelligent robotic control.

Although considerable progress has been achieved, existing NN-based control schemes still face two major challenges when applied to complex robotic systems. On the one hand, most adaptive NN control methods can only guarantee Uniformly Ultimately Bounded (UUB) tracking performance rather than true asymptotic convergence of the tracking error. This implies that the system states converge merely to a finite neighborhood of the origin instead of approaching zero strictly, which limits their applicability in high-precision trajectory tracking tasks. On the other hand, the majority of existing approaches rely on feedforward neural network architectures, such as the RBFNN [12,13]. These networks provide only static input–output mappings and thus lack the capability to capture time-varying internal dynamics inherent to robotic systems [14,15]. Consequently, their approximation performance deteriorates in dynamically changing environments. Furthermore, RBFNN-based controllers often depend on offline training and manual parameter tuning, leading to slow adaptation, poor generalization, and degraded control accuracy under time-varying conditions [12]. Although some recent control methods in the literature are based on echo state networks, a type of recurrent neural network, these methods adopt random or complicated network topologies, leading to high computational complexity and limited practical applicability in real robotic systems [16,17].

The primary objective of this work is to bridge the gap between tracking precision and computational efficiency in the control of highly uncertain, dynamic MIMO robotic systems, thereby overcoming the limitations of conventional UUB tracking performance and high network complexity. To achieve this objective, a novel neuroadaptive asymptotic tracking control strategy is proposed by integrating an intrinsic plasticity-driven cycle echo state network with the robust integral of the sign of the error framework. Compared with existing control methods in the literature, the main contributions of this work are threefold:

- First, a novel recurrent reservoir featuring a simplified cycle structure and biological learning rules is constructed to enhance representation capability while minimizing computational overhead. Unlike conventional ESNs that utilize random or complex reservoir topologies which heavily increase the computational burden and limit real-time applicability, the reservoir in our scheme adopts a deterministic, simple cycle structure. This design significantly reduces the density of neural interconnections, effectively alleviating the computational burden and lowering algorithmic complexity to facilitate faster real-time calculation. Furthermore, to prevent any degradation in approximation performance due to the simplified structure, an intrinsic plasticity mechanism—a foundational biological learning rule—is embedded for online tuning of the excitability of each reservoir neuron [18,19]. This drastically improves the reservoir’s dynamic representation capability to accurately capture the highly coupled, complex, and time-varying internal nonlinearities inherent in robotic systems.
- Second, a composite neuroadaptive controller is synthesized to achieve robust approximation of unknown dynamics and disturbance rejection capabilities. The controller is developed by employing the proposed IP-driven cycle ESN to dynamically approximate unknown system nonlinearities, while leveraging the RISE framework to continuously compensate for residual approximation errors and external time-varying disturbances, thereby guaranteeing high-precision tracking.
- Third, a rigorous theoretical proof and two simulation scenarios are provided. A comprehensive stability analysis based on Lyapunov theory is carried out to provide a solid mathematical guarantee for the asymptotic stability of the closed-loop system. Furthermore, two simulations are conducted on typical two-joint manipulators to evaluate the performance of the proposed method over conventional RBFNN-based strategies in balancing tracking precision, convergence speed, and control smoothness.

It should be noted that the scope of this work is limited to numerical simulation studies. The proposed control strategy is validated only under smooth continuous reference trajectories. Practical issues, including experimental validation and trajectory tracking under abrupt or discontinuous reference trajectories, are beyond the scope of this paper and will be investigated in future work.

The remainder of this manuscript is structured as follows. Section 2 establishes the problem formulation of the uncertain multi-joint robotic systems and briefly reviews the preliminaries regarding the topological architecture and universal approximation capability of the proposed IP-driven cycle ESN. Section 3 presents the detailed design procedure of the composite neuroadaptive controller and its corresponding weight update laws, followed by a rigorous mathematical proof of the closed-loop stability and asymptotic tracking performance. In Section 4, two numerical simulation scenarios are executed on different two-joint robotic manipulators to demonstrate the effectiveness and comparative advantages of the developed scheme. Section 5 provides a thorough discussion of the simulation results and the underlying limitations. Finally, Section 6 concludes the paper with a summary of key findings and prospects for future research.

## 2. Problem Formulation and Preliminaries

### 2.1. Problem Formulation

Consider a class of fully actuated n-link rigid robotic systems modeled via the classical Euler–Lagrange formula [20]:

(1) $$M\left(q\right)\ddot{q}+C\left(q,\dot{q}\right)\dot{q}+G\left(q\right)+F\left(\dot{q}\right)+\tau_{d}\left(t\right)=u$$

where $q\in R^{n}$ is the joint angular displacement vector, $M\left(q\right)\in R^{n\times n}$ is the inertia matrix, $C\left(q,\dot{q}\right)\in R^{n\times n}$ denotes the centripetal and Coriolis terms, $G\left(q\right)\in R^{n}$ represents the gravitational vector, $F\left(\dot{q}\right)\in R^{n}$ signifies the friction force, $\tau_{d}\left(t\right)\in R^{n}$ is the unknown external disturbance, and $u\in R^{n}$ is the control input. To facilitate the controller design, the inherent structural properties of the dynamic model (1) must be considered. Specifically, the inertia matrix M(q) is symmetric and positive-definite, satisfying $m_{1}{∥x∥}^{2}\leq x^{T}M\left(q\right)x\leq m_{2}{∥x∥}^{2}$ for any arbitrary vector $x\in R^{n}$, where $m_{1}$ and $m_{2}$ are positive constants. In the subsequent stability analysis, this specific property provides a solid foundation to guarantee the positive-definiteness of the formulated Lyapunov function.

A neuroadaptive asymptotic tracking control strategy is proposed in this paper to ensure that: ① the joint angular displacement *q* of system (1) asymptotically tracks the desired trajectory $q_{d}$; and ② all signals are bounded in the closed-loop system.

To achieve the above objectives, the following assumptions are made.

**Assumption** **1.** *The joint angular displacement q and joint angular velocity* $\dot{q}$ *of system (1) are available for measurement.*

**Assumption** **2.** *The desired trajectory* $q_{d}$ *and its first, second and third derivatives are known, bounded and continuous functions.*

**Assumption** **3.** *The system matrices* M(q)*,* $C(q,\dot{q})$*,* G(q)*, and* $F(\dot{q})$ *are assumed to be smooth. Meanwhile, the external disturbance* $\tau_{d}\left(t\right)$ *and its first two time derivatives are bounded by some constants* $\varepsilon_{\tau 0}$*,* $\varepsilon_{\tau 1}$*,* $\varepsilon_{\tau 2}$*, satisfying* $∥\tau_{d}∥\leq \varepsilon_{\tau 0}$*,* $∥{\dot{\tau }}_{d}∥\leq \varepsilon_{\tau 1}$*, and* $∥{\ddot{\tau }}_{d}∥\leq \varepsilon_{\tau 2}$*.*

Hereafter, for the sake of simplicity, the function arguments are occasionally omitted when there is no risk of ambiguity, that is: f(t) refers to any function vector of *t* and *f* is the same to f(t).

Then, define the tracking error $e_{1}$ and the filtering error *r*

(2) $$e_{1}=q_{d}-q$$

(3) $$e_{2}={\dot{e}}_{1}+\alpha_{1}e_{1}$$

(4) $$r={\dot{e}}_{2}+\alpha_{2}e_{2}$$

where $\alpha_{1}>0.5$ and $\alpha_{2}>0.5$ are design parameters.

By multiplying both sides of Equation (4) by M(q), and then substituting Equations (2) and (3), it can be found that

(5) $$\begin{array}{cc} M(q)r= & M({\ddot{e}}_{1}+\alpha_{1}{\dot{e}}_{1}+\alpha_{2}e_{2})\\ = & M(\alpha_{1}{\dot{e}}_{1}+\alpha_{2}e_{2}+{\ddot{q}}_{d})\\ \; & +C\left(q,\dot{q}\right)\dot{q}+G\left(q\right)+F\left(\dot{q}\right)+\tau_{d}-u\\ = & Q\left(\cdot \right)-u \end{array}$$

where $Q\left(\cdot \right)=M\left(q\right)\left(\alpha_{1}{\dot{e}}_{1}+\alpha_{2}e_{2}+{\ddot{q}}_{d}\right)+C\left(q,\dot{q}\right)\dot{q}+G\left(q\right)+F\left(\dot{q}\right)+\tau_{d}$ lumps all the model-dependent terms.

Differentiating Equation (5) with respect to time gives

$$M\left(q\right)\dot{r}=-\dot{M}\left(q\right)r-\dot{u}+\dot{Q}\left(\cdot \right).$$

By splitting the term $-\dot{M}\left(q\right)r$ into two equal parts and adding and subtracting $e_{2}$, the above equation can be rewritten as

(6) $$\begin{array}{cc} M\dot{r} & =-\frac{1}{2}\dot{M}r-\dot{u}-e_{2}+\dot{Q}\left(\cdot \right)-\frac{1}{2}\dot{M}r+e_{2}\\ \; & =-\frac{1}{2}\dot{M}r-\dot{u}-e_{2}+N\left(\cdot \right) \end{array}$$

where $N\left(\cdot \right)=\dot{Q}\left(\cdot \right)-\frac{1}{2}\dot{M}r+e_{2}$ represents the remaining unknown time-varying terms that can be approximated by the neural network as illustrated in Section 2.2.

### 2.2. Intrinsic Plasticity-Driven Cycle Echo State Network and Its Approximating Capability

In Equation (6), the term N(·) includes the inertia matrix M(q), the Coriolis and centrifugal force term $C(q,\dot{q})$, the gravitational vector G(q), the friction force $F(\dot{q})$ and the external disturbance $\tau_{d}$. These unknown terms, as they cannot be accurately modeled or compensated by traditional control methods, make the control task challenging. To address this challenge, in this paper, an intrinsic plasticity-driven cycle echo state network is proposed to approximate these complex and time-varying nonlinear functions. In this structure, the cycle reservoir topology significantly reduces the number of connections, while the intrinsic plasticity rule online adapts each neuron’s gain and bias parameters. This combination not only enhances the reservoir’s dynamic representation capability but also improves computational efficiency, making it particularly suitable for real-time robotic control under uncertainties.

**Figure 1:**
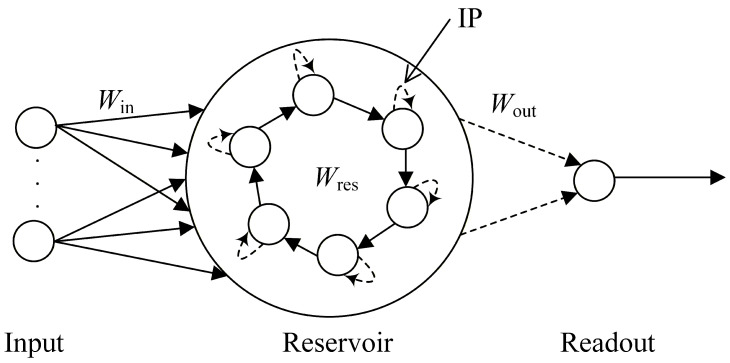
The architecture of the IP-driven cycle ESN.

The architecture of the proposed IP-driven cycle ESN is illustrated in Figure 1. The network structure consists of three components: input layer, reservoir and readout layer. The computational procedure is as follows:

(7) $$\begin{array}{cc} h_{in}\left(t\right) & =W_{in}^{T}z\left(t\right)+W_{res}^{T}h\left(t-1\right) \end{array}$$

(8) $$\begin{array}{cc} h(t) & =\phi \left(a_{j}\left(t\right)h_{in}\left(t\right)+b_{j}\left(t\right)\right) \end{array}$$

(9) $$\begin{array}{cc} y_{out}\left(t\right) & =W_{out}^{T}h\left(t\right) \end{array}$$

(10) $$\begin{array}{cc} a_{j}\left(t+1\right) & =a_{j}\left(t\right)+\frac{\eta }{a_{j}\left(t\right)}+h_{in_{j}}\left(t\right)\Delta b_{j}\left(t\right) \end{array}$$

(11) $$\begin{array}{cc} b_{j}\left(t+1\right) & =b_{j}\left(t\right)+\eta \left(1-\left(2+\frac{1}{\mu }\right)h_{j}\left(t\right)+\frac{1}{\mu }h_{j}^{2}\left(t\right)\right) \end{array}$$

where $z\left(t\right)\in R^{A\times 1}$ is the input of the ESN, $h_{in}\left(t\right)\in R^{B\times 1}$ is the input of the neurons in the reservoir, $h\left(t\right)\in R^{B\times 1}$ is the reservoir output state vector at the current moment, $W_{in}\in R^{A\times B}$ is the weight from input to the reservoir, $W_{res}\in R^{B\times B}$ is the weight of the reservoir layer, $W_{out}\in R^{B\times C}$ is the weight from the reservoir to the readout and $y_{out}\in R^{C\times 1}$ is the ESN output. The variables *A*, *B* and *C* denote the neuron counts in the input, reservoir and readout components, respectively. The function ϕ is the activation function of the reservoir component, and the sigmoid function is adopted in this work. Therefore, it yields $∥\phi ∥\leq \varepsilon_{\phi 0}$ and $∥\dot{\phi }∥\;\leq \varepsilon_{\phi 1}$, where $\varepsilon_{\phi 0}$ and $\varepsilon_{\phi 1}$ are some positive constants. Parameters $a_{j}\left(t\right)$ and $b_{j}\left(t\right)$ represent the gain and bias of the activation function of the *j*-th neuron in the reservoir at time *t*, respectively. μ is the expected distribution mean, η is the intrinsic plasticity learning rate, $h_{in_{j}}\left(t\right)$ is the input to the activation function of the *j*-th neuron in the reservoir at time *t*, $h_{j}\left(t\right)$ is the reservoir output of the *j*-th neuron in the reservoir, and $\Delta b_{j}\left(t\right)=b_{j}\left(t\right)-b_{j}\left(t-1\right)$. In this paper, the readout weight matrix $W_{out}$ is obtained by the adaptive laws, while the input layer weight matrix $W_{in}$ and the reservoir weight matrix $W_{res}$ are set as follows:

$$W_{in}={\left[\begin{array}{cccc} \nu & \nu & \cdots & \nu \\ \nu & \nu & \cdots & \nu \\ \vdots & \vdots & \ddots & \vdots \\ \nu & \nu & \cdots & \nu \end{array}\right]}_{A\times B},W_{res}={\left[\begin{array}{cccc} 0 & \cdots & 0 & \rho \\ \rho & \cdots & 0 & 0\\ \vdots & \ddots & \vdots & \vdots \\ 0 & \cdots & \rho & 0 \end{array}\right]}_{B\times B}$$

where ν and ρ are some positive constants. It is worth mentioning that unlike conventional echo state networks that suffer from heavy computational burdens due to randomized or fully connected topologies, the proposed deterministic cycle structure establishes a highly decoupled and sparse state propagation mechanism. This structural simplification directly translates into a significant reduction in algorithmic complexity during the online control process. To further illustrate the advantages of this design from a computational perspective, the following remark is provided.

**Remark** **1.** *Let N denote the number of internal neurons within the reservoir. For a fully connected reservoir, the internal weight matrix* $W_{res}\in R^{N\times N}$ *is dense. Updating reservoir states involves full matrix-vector multiplication, leading to a quadratic computational complexity of *$O(N^{2})$* per control step. In contrast, the cycle reservoir adopts a deterministic cycle topology with a highly sparse weight matrix, where non-zero elements only lie on the sub-diagonal and the corner entry. Benefiting from this cycle connection, the state of each neuron is only related to its adjacent neuron. The original matrix-vector multiplication is simplified into N scalar operations, and the computational complexity is reduced to *O(N)* per control step. Meanwhile, the number of effective connections decreases from *$N^{2}$* to N. This significant reduction greatly lowers the computational overhead, making the proposed algorithm well applicable to high-frequency real-time robotic control under limited hardware resources.*

According to the Universal Approximation Theorem for neural networks, given any compact set $\Omega_{N}\in R$, for all $z\in \Omega_{N}$, N(·) can be presented by the ESN as

(12) $$N\left(\cdot \right)=W^{T}\phi \left(z\right)+\varepsilon$$

where *W* is the output weight and ε is approximation error, which satisfies the assumption that $∥\varepsilon ∥\leq \varepsilon_{n0}$, $\varepsilon_{n0}$ is an unknown positive constant. Moreover, in this work, it is assumed that $∥\dot{\varepsilon }∥\leq \varepsilon_{n1}$, $\varepsilon_{n1}$ is an unknown positive constant.

Then Equation (6) can be re-expressed as

(13) $$M\left(q\right)\dot{r}=-\frac{1}{2}\dot{M}\left(q\right)r+W^{T}\phi \left(z\right)+\varepsilon -\dot{u}-e_{2}.$$

It is worth mentioning that as *W* is unknown, then the approximation of N(·) by ESN can be redefined as

(14) $$\hat{N}\left(\cdot \right)={\hat{W}}^{T}\phi \left(z\right)$$

where $z\left(t\right)={\left[q^{T}\left(t\right),\;{\dot{q}}^{T}\left(t\right),\;q_{d}^{T}\left(t\right),\;{\dot{q}}_{d}^{T}\left(t\right),\;{\ddot{q}}_{d}^{T}\left(t\right),\;e_{1}^{T}\left(t\right),\;e_{2}^{T}\left(t\right),\;{\dot{e}}_{1}^{T}\left(t\right)\right]}^{T}\in R^{8n}$, $\hat{W}$ is the estimated value of *W*.

## 3. Controller Design and Stability Analysis

**Figure 2:**
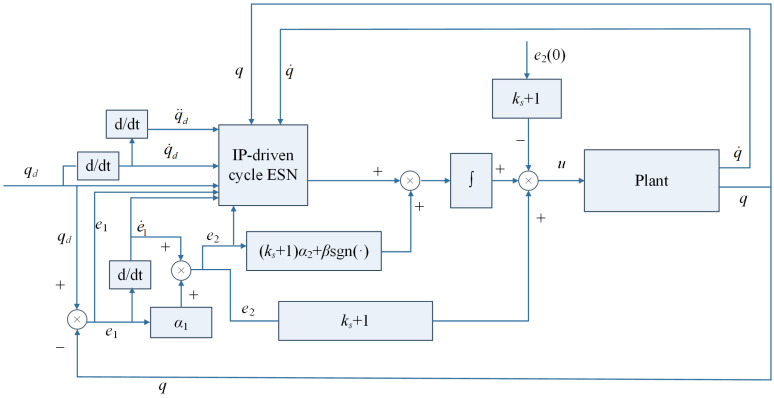
Overall architecture of the proposed control scheme.

Based on the system dynamics in (13), the control scheme is given by

(15) $$u=\left(k_{s}+1\right)e_{2}\left(t\right)-\left(k_{s}+1\right)e_{2}\left(0\right)+\int_{0}^{t}\left[\left(k_{s}+1\right)\alpha_{2}e_{2}\left(\tau \right)+\beta \mathrm{sgn}\left(e_{2}\left(\tau \right)\right)+{\hat{W}}^{T}\phi \left(z\left(\tau \right)\right)\right]d\tau$$

where $k_{s}$ and β are some positive constants. The overall architecture of the proposed controller is illustrated in Figure 2.

The corresponding adaptive law for $\hat{W}$ is given as

(16) $$\dot{\hat{W}}=k_{n}\alpha_{2}\phi e_{2}^{T}-k_{w}k_{n}∥e_{2}∥\hat{W}$$

where $k_{n}$ and $k_{w}$ are positive designed parameters. The first term $k_{n}\alpha_{2}\phi e_{2}^{T}$ is used for neural compensation of the error dynamics, while the second term $-k_{w}k_{n}∥e_{2}∥\hat{W}$ is introduced to prevent parameter drift and ensure boundedness of the weight estimates under approximation errors and external disturbances.

Substitute Equation (15) into Equation (13), and the close-loop tracking dynamics of the system can be obtained:

(17) $$M\left(q\right)\dot{r}=-\frac{1}{2}\dot{M}\left(q\right)r-\left(k_{s}+1\right)r-\beta \mathrm{sgn}\left(e_{2}\right)-{\tilde{W}}^{T}\phi +\varepsilon -e_{2}$$

where $\tilde{W}=\hat{W}-W$ is the weight estimation error.

To facilitate the stability analysis, the following Lemma 1 and Lemma 2 are introduced.

**Lemma** **1.** *Give an auxiliary function* L(t) *defined as*

(18) $$L\left(t\right)=r^{T}\left(\varepsilon -\beta \mathrm{sgn}\left(e_{2}\right)\right)+\frac{1}{4}k_{w}W_{n}^{2}e_{2}^{T}\mathrm{sgn}\left(e_{2}\right)-{\dot{e}}_{2}^{T}H$$

*where* $W_{n}$ *is the upper bound of* $∥W∥$ *,* $H={\tilde{W}}^{T}\phi$ *. H and its time derivative* $\dot{H}$ *are bounded [21]. Specifically, there exist positive constants* $\varepsilon_{h0}$ *and* $\varepsilon_{h1}$ *satisfying* $∥H∥\leq \varepsilon_{h0}$ *and* $∥\dot{H}∥\leq \varepsilon_{h1}$ *. Provided that the control gain β is chosen to fulfill the following sufficient condition,*

(19) $$\beta >\varepsilon_{n0}+\max\left\{\varepsilon_{h0},\frac{1}{4\alpha_{2}}k_{w}W_{n}^{2}+\frac{1}{\alpha_{2}}\varepsilon_{n1}+\frac{1}{\alpha_{2}}\varepsilon_{h1}\right\}$$

*then it follows that*

(20) $$\int_{0}^{t}L(\tau )d\tau \leq \xi$$

*where* $\xi =\beta {∥e_{2}\left(0\right)∥}_{1}-e_{2}^{T}\left(0\right)\left(\varepsilon \left(0\right)-H\left(0\right)\right)$ *.*

**Proof.**  After expanding L(t) and integrating, it is observed that

(21) $$\begin{array}{cc} \int_{0}^{t}L(\tau )d\tau = & \int_{0}^{t}r^{T}\left(\tau \right)\left(\varepsilon \left(\tau \right)-\beta \mathrm{sgn}(e_{2}\left(\tau \right))\right)d\tau +\frac{1}{4}k_{w}W_{n}^{2}\int_{0}^{t}e_{2}^{T}\left(\tau \right)\mathrm{sgn}\left(e_{2}\left(\tau \right)\right)d\tau \\ \; & -\int_{0}^{t}{\dot{e}}_{2}^{T}\left(\tau \right)H\left(\tau \right)d\tau \end{array}$$

Substituting (4) into the above equation and rearranging terms leads to

(22) $$\begin{array}{cc} \int_{0}^{t}L(\tau )d\tau = & \int_{0}^{t}\alpha_{2}{e_{2}}^{T}\left(\tau \right)\left(\varepsilon \left(\tau \right)-\beta \mathrm{sgn}(e_{2}\left(\tau \right))\right)d\tau +\int_{0}^{t}{\dot{e}}_{2}^{T}\left(\tau \right)\left(\varepsilon \left(\tau \right)-\beta \mathrm{sgn}(e_{2}\left(\tau \right))\right)d\tau \\ \; & +\frac{1}{4}k_{w}W_{n}^{2}\int_{0}^{t}e_{2}^{T}\left(\tau \right)\mathrm{sgn}\left(e_{2}\left(\tau \right)\right)d\tau -\int_{0}^{t}{\dot{e}}_{2}^{T}\left(\tau \right)H\left(\tau \right)d\tau \\ = & \int_{0}^{t}\alpha_{2}{e_{2}}^{T}\left(\tau \right)\left(\varepsilon \left(\tau \right)-\beta \mathrm{sgn}\left(e_{2}\left(\tau \right)\right)+\frac{1}{4\alpha_{2}}k_{w}W_{n}^{2}\mathrm{sgn}\left(e_{2}\left(\tau \right)\right)\right)d\tau \\ \; & +\int_{0}^{t}{\dot{e}}_{2}^{T}\left(\tau \right)\left(\varepsilon \left(\tau \right)-H\left(\tau \right))-\beta \mathrm{sgn}\left(e_{2}\left(\tau \right)\right)\right)d\tau \end{array}$$

By applying integration by parts to the second term of the above equation, it follows that

(23) $$\begin{array}{cc} \int_{0}^{t}L(\tau )d\tau = & \int_{0}^{t}\alpha_{2}{e_{2}}^{T}\left(\tau \right)\left(\varepsilon \left(\tau \right)-\beta \mathrm{sgn}\left(e_{2}\left(\tau \right)\right)+\frac{1}{4\alpha_{2}}k_{w}W_{n}^{2}\mathrm{sgn}\left(e_{2}\left(\tau \right)\right)\right)d\tau \\ \; & +\left(e_{2}^{T}\left(\tau \right)\left(\varepsilon \left(\tau \right)-H\left(\tau \right)\right)\right){|}_{0}^{t}-\int_{0}^{t}\left(e_{2}^{T}\left(\tau \right)\dot{\varepsilon }\left(\tau \right)-e_{2}^{T}\left(\tau \right)\dot{H}\left(\tau \right)\right)d\tau -\beta \int_{0}^{t}{\dot{e}}_{2}^{T}\mathrm{sgn}\left(e_{2}\right)d\tau \\ = & \int_{0}^{t}\alpha_{2}e_{2}^{T}\left(\tau \right)\left(\varepsilon \left(\tau \right)-\beta \mathrm{sgn}\left(e_{2}\left(\tau \right)\right)+\frac{1}{4\alpha_{2}}k_{w}W_{n}^{2}\mathrm{sgn}\left(e_{2}\left(\tau \right)\right)\right)d\tau \\ \; & +e_{2}^{T}\left(t\right)\left(\varepsilon (t)-H(t)\right)-e_{2}^{T}\left(0\right)\left(\varepsilon (0)-H(0)\right)\\ \; & -\int_{0}^{t}\left(e_{2}^{T}\left(\tau \right)\dot{\varepsilon }\left(\tau \right)-e_{2}^{T}\left(\tau \right)\dot{H}\left(\tau \right)\right)d\tau -\beta ∥e_{2}{\left(t\right)∥}_{1}+\beta {∥e_{2}\left(0\right)∥}_{1} \end{array}$$

Utilizing the Cauchy–Schwarz inequality and the relationship $∥\cdot ∥\leq {∥\cdot ∥}_{1}$, and further collecting like terms, it follows that

(24) $$\begin{array}{cc} \int_{0}^{t}L\left(\tau \right)d\tau \leq & \int_{0}^{t}\alpha_{2}∥e_{2}\left(\tau \right)∥\left(∥\varepsilon \left(\tau \right)∥-\beta +\frac{1}{4\alpha_{2}}k_{w}W_{n}^{2}+\frac{∥\dot{\varepsilon }\left(\tau \right)∥}{\alpha_{2}}+\frac{∥\dot{H}\left(\tau \right)∥}{\alpha_{2}}\right)\;d\tau \\ \; & +∥e_{2}{\left(t\right)∥}_{1}\left(∥\varepsilon (t)∥+∥H(t)∥-\beta \right)-e_{2}^{T}\left(0\right)\left(\varepsilon (0)-H(0)\right)+\beta {∥e_{2}\left(0\right)∥}_{1} \end{array}$$

Given $∥\varepsilon ∥\leq \varepsilon_{n0}$, $∥\dot{\varepsilon }∥\leq \varepsilon_{n1}$, $∥H∥\leq \varepsilon_{h0}$, and $∥\dot{H}∥\leq \varepsilon_{h1}$ it follows that:

(25) $$\begin{array}{cc} \int_{0}^{t}L\left(\tau \right)d\tau \leq & \int_{0}^{t}\alpha_{2}∥e_{2}\left(\tau \right)∥\left(\varepsilon_{n0}-\beta +\frac{1}{4\alpha_{2}}k_{w}W_{n}^{2}+\frac{\varepsilon_{n1}}{\alpha_{2}}+\frac{\varepsilon_{h1}}{\alpha_{2}}\right)\;d\tau \\ \; & +∥e_{2}{\left(t\right)∥}_{1}\left(\varepsilon_{n0}+\varepsilon_{h0}-\beta \right)-e_{2}^{T}\left(0\right)\left(\varepsilon (0)-H(0)\right)+\beta {∥e_{2}\left(0\right)∥}_{1} \end{array}$$

Finally, when β is chosen to satisfy (19), inequality (20) follows directly. □

**Lemma** **2** ([22])**.** *Consider the nonlinear time-varying system*

(26) $$\dot{\zeta }=f\left(\zeta ,t\right),$$

*where *$\zeta \in R^{m}$* and *$f:R^{m}\times R_{\geq 0}\to R^{m}$* is continuous, ensuring the existence of a unique solution. Define the region*

(27) $$D:=\left\{\zeta \in R^{m}:∥\zeta ∥<\varsigma \right\},$$

*where ς is a positive constant. Suppose that there exists a continuously differentiable function *$V:D\times R_{\geq 0}\to R_{\geq 0}$* satisfying*

(28) $$U_{1}\left(\zeta \right)\leq V\left(\zeta ,t\right)\leq U_{2}\left(\zeta \right),\;\dot{V}\left(\zeta ,t\right)\leq -U\left(\zeta \right),$$

*for all *t≥0* and *ζ∈D*, where *$U_{1}$* and *$U_{2}$* are continuous positive-definite functions, and U is a uniformly continuous positive-semidefinite function. Then, for every initial condition *ζ(0)* belonging to*

(29) $$S:=\left\{\zeta \in D:U_{2}\left(\zeta \right)\leq \delta \right\},\;\mathrm{where}\;\delta <\min_{∥\zeta ∥=\varsigma }U_{1}\left(\zeta \right),$$

*it follows that*

(30) U(ζ(t))→0ast→∞.

*Now we are prepared to introduce the subsequent result concerning the neuroadaptive control strategy for the robotic manipulators.*

**Theorem** **1.** *Consider the system (1) with error dynamics described by (2)–(4). Suppose that Assumptions 1–3 hold. If the control law is designed as (15) together with the ESN weight update law (16), then all system signals are bounded, and the tracking error converges to zero asymptotically,* $∥e_{1}\left(t\right)∥\to 0$ *as* t→∞ *.*

**Proof.**  Consider the following Lyapunov function candidate:

(31) $$V\left(y,t\right)=\frac{1}{2}r^{T}Mr+Q+\frac{1}{2}e_{1}^{T}e_{1}+\frac{1}{2}e_{2}^{T}e_{2}+P\left(t\right)$$

where $Q=\frac{1}{2k_{n}}\mathrm{tr}\left({\tilde{W}}^{T}\tilde{W}\right)$. The auxiliary non-negative function P(t) is defined as follows:

(32) $$P\left(t\right)=\xi -\int_{0}^{t}L(\tau )d\tau$$

where ξ and *L* are defined in (18)–(20). It can be verified that P(t)≥0. And y(t) is defined as

(33) $$y\left(t\right):={\left[\begin{array}{ccccc} r^{T}\left(t\right) & e_{1}^{T}\left(t\right) & e_{2}^{T}\left(t\right) & \sqrt{Q} & \sqrt{P} \end{array}\right]}^{T},$$

According to the positive definiteness of the inertia matrix M(q), there exist positive constants $m_{1}$ and $m_{2}$ such that

(34) $$m_{1}{∥r∥}^{2}\leq r^{T}M\left(q\right)r\leq m_{2}{∥r∥}^{2}.$$

Therefore, the Lyapunov function can be bounded as

(35) $$\lambda_{1}{∥y∥}^{2}\leq V\left(y,t\right)\leq \lambda_{2}{∥y∥}^{2},$$

where

(36) $$\lambda_{1}:=\frac{1}{2}\min\left\{m_{1},1\right\},\;\lambda_{2}:=\max\left\{\frac{1}{2}m_{2},1\right\}.$$

Differentiating V(t) with respect to time yields

(37) $$\dot{V}\left(t\right)=r^{T}M\dot{r}+\frac{1}{2}r^{T}\dot{M}r+\frac{1}{k_{n}}\mathrm{tr}\left({\tilde{W}}^{T}\dot{\tilde{W}}\right)+e_{1}^{T}{\dot{e}}_{1}+e_{2}^{T}{\dot{e}}_{2}+\dot{P}$$

Substituting (16)–(18) into $\dot{V}\left(t\right)$ results in

(38) $$\dot{V}\left(t\right)=-\left(k_{s}+1\right)r^{T}r-k_{w}∥e_{2}∥\mathrm{tr}\left({\tilde{W}}^{T}\hat{W}\right)+e_{1}^{T}e_{2}-\alpha_{1}e_{1}^{T}e_{1}-\alpha_{2}e_{2}^{T}e_{2}-k_{w}\frac{W_{n}^{2}}{4}{e_{2}}^{T}\mathrm{sgn}\left(e_{2}\right)$$

Furthermore, noting that $\hat{W}=\tilde{W}+W$, yielding

(39) $$\begin{array}{cc} -\mathrm{tr}({\tilde{W}}^{T}\hat{W}) & =-\mathrm{tr}\left({\tilde{W}}^{T}\left(\tilde{W}+W\right)\right)\\ \; & =-∥\tilde{W}{∥}^{2}-\mathrm{tr}\left({\tilde{W}}^{T}W\right)\\ \; & \leq -∥\tilde{W}{∥}^{2}+∥\tilde{W}∥∥W∥\\ \; & =-{\left(∥\tilde{W}∥-\frac{∥W∥}{2}\right)}^{2}+\frac{1}{4}{∥W∥}^{2} \end{array}$$

Next, by invoking Young’s inequality, it follows that

(40) $$e_{1}^{T}e_{2}\leq \frac{1}{2}e_{1}^{T}e_{1}+\frac{1}{2}e_{2}^{T}e_{2}$$

By combining like terms with $-\alpha_{1}e_{1}^{T}e_{1}-\alpha_{2}e_{2}^{T}e_{2}$, the grouped terms $-\left(\alpha_{1}-\frac{1}{2}\right)e_{1}^{T}e_{1}$ and $-\left(\alpha_{2}-\frac{1}{2}\right)e_{2}^{T}e_{2}$ are obtained. Furthermore, from $∥W∥\leq W_{n}$, it follows that

(41) $$\begin{array}{cc} \dot{V}\left(t\right) & \leq -\left(k_{s}+1\right)r^{T}r-\left(\alpha_{1}-\frac{1}{2}\right)e_{1}^{T}e_{1}-\left(\alpha_{2}-\frac{1}{2}\right)e_{2}^{T}e_{2}-k_{w}∥e_{2}∥{\left(∥\tilde{W}∥-\frac{∥W∥}{2}\right)}^{2}\\ \; & \;+\frac{1}{4}k_{w}∥e_{2}∥{∥W∥}^{2}-\frac{1}{4}k_{w}{∥e_{2}∥}_{1}W_{n}^{2}\\ \; & \leq -\left(k_{s}+1\right)r^{T}r-\left(\alpha_{1}-\frac{1}{2}\right)e_{1}^{T}e_{1}-\left(\alpha_{2}-\frac{1}{2}\right)e_{2}^{T}e_{2} \end{array}$$

Consequently, $\dot{V}\left(t\right)$ can be upper-bounded as

(42) $$\dot{V}\left(t\right)\leq -U\left(y\right)$$

where

$$U\left(y\right)=\left(k_{s}+1\right)r^{T}r+\left(\alpha_{1}-\frac{1}{2}\right)e_{1}^{T}e_{1}+\left(\alpha_{2}-\frac{1}{2}\right)e_{2}^{T}e_{2}.$$

Since $k_{s}>0$, $\alpha_{1}>1/2$, and $\alpha_{2}>1/2$, as specified in (4) and (15), U(y) is a positive semidefinite function. From Equations (31) and (42), it is straightforward to see that $V\left(t\right)\in L_{\infty }$. From the definition of V(t), we can conclude that the signals *r*, $e_{1}$, $e_{2}$, $\tilde{W}$ (and thus $\hat{W}$), and *P* are all bounded. According to Equations (3) and (4) and (15)–(17), this further guarantees the boundedness of ${\dot{e}}_{1}$, $\dot{e_{2}}$, $\dot{\hat{W}}\left(\dot{\tilde{W}}\right)$, $\dot{r}$, and the control input *u*. Therefore, all closed-loop signals remain bounded.

Furthermore, define

$$D:=\{y\in R^{3n+2}:∥y∥<\varsigma \},$$

where ς>0 is sufficiently large such that the y(t) remains inside *D* for all t≥0, which follows from the boundedness of all closed-loop signals established above. Correspondingly, let

$$S:=\left\{y\in D:U_{2}\left(y\right)\leq \delta \right\},$$

where

$$\delta <\min_{∥y∥=\varsigma }U_{1}\left(y\right).$$

Then, from (35), the Lyapunov function satisfies

$$U_{1}\left(y\right)\leq V\left(y,t\right)\leq U_{2}\left(y\right),$$

where $U_{1}\left(y\right)=\lambda_{1}{∥y∥}^{2}$ and $U_{2}\left(y\right)=\lambda_{2}{∥y∥}^{2}$ are continuous positive-definite functions. Moreover, (42) gives $\dot{V}\left(y,t\right)\leq -U\left(y\right),$ where U(y) is a continuous positive semidefinite function. Since all closed-loop signals have been shown to be bounded, the y(t) evolves in the compact set *S*, where U(y) is uniformly continuous. Therefore, according to Lemma 2, it follows that

$$\lim_{t\to \infty }U\left(y\left(t\right)\right)=0.$$

Since $k_{s}+1>0$, $\alpha_{1}>1/2$, and $\alpha_{2}>1/2$, it follows that

$$\lim_{t\to \infty }∥r\left(t\right)∥=0,\;\lim_{t\to \infty }∥e_{1}\left(t\right)∥=0,\;\lim_{t\to \infty }∥e_{2}\left(t\right)∥=0.$$

Hence, the closed-loop system is asymptotically stable. □

Based on the rigorous stability analysis and the derived control laws, the detailed real-time implementation procedure of the proposed control scheme is systematically summarized in Table 1.

## 4. Simulation

In this section, two simulation scenarios are provided to verify the effectiveness and superiority of the designed control scheme. The entire simulation framework is established and carried out utilizing MATLAB R2023b on a personal computer equipped with a 64-bit Windows 11 Professional operating system, an AMD Ryzen 7 7435H CPU@3.10 GHz, and 40 GB of system memory. The specific dynamic parameters, initial states, and reference trajectories of the manipulator system are systematically provided in each scenario.

- Simulation Scenario 1

In this scenario, the simulation is performed on the following two-joint robotic system [23].

$$\left[\begin{array}{cc} M_{11} & M_{12}\\ M_{12} & M_{22} \end{array}\right]\left[\begin{array}{c} {\ddot{q}}_{1}\\ {\ddot{q}}_{2} \end{array}\right]+\left[\begin{array}{cc} C_{11} & C_{12}\\ C_{21} & C_{22} \end{array}\right]\left[\begin{array}{c} {\dot{q}}_{1}\\ {\dot{q}}_{2} \end{array}\right]+\left[\begin{array}{c} G_{1}\\ G_{2} \end{array}\right]+\left[\begin{array}{c} F_{1}\\ F_{2} \end{array}\right]+\tau_{d}=u$$

where $M_{11}=\left(m_{1}+m_{2}\right)r_{1}^{2}+m_{2}r_{2}^{2}+2m_{2}r_{1}r_{2}\cos\left(q_{2}\right)+J_{1}$, $M_{12}=m_{2}r_{2}^{2}+m_{2}r_{1}r_{2}\cos\left(q_{2}\right)$, $M_{22}=m_{2}r_{2}^{2}+J_{2}$, $C_{11}=-m_{2}r_{1}r_{2}\sin\left(q_{2}\right){\dot{q}}_{2}$, $C_{12}=-2m_{2}r_{1}r_{2}\sin\left(q_{2}\right)\left({\dot{q}}_{1}+{\dot{q}}_{2}\right)$, $C_{21}=0$, $C_{22}=m_{2}r_{1}r_{2}\sin\left(q_{2}\right){\dot{q}}_{2}$, $G_{1}=\left(m_{1}+m_{2}\right)gr_{1}\cos\left(q_{1}\right)+m_{2}gr_{2}\left(q_{1}+q_{2}\right)$, $G_{2}=m_{2}gr_{2}\cos\left(q_{1}+q_{2}\right)$, $F_{1}=0.5{\dot{q}}_{1}+0.3\sin\left({\dot{q}}_{1}\right)$, $F_{2}=0.3{\dot{q}}_{2}-0.4\sin\left({\dot{q}}_{2}\right)$. $m_{1}$ and $m_{2}$ are the masses of the two joints, $r_{1}$ and $r_{2}$ are the lengths of the joints and *g* represents the gravitational constant. In this work, the system parameters are chosen as $m_{1}=0.5$ kg, $m_{2}=1.5$ kg, $r_{1}=1.0$ m, $r_{2}=0.8$ m, $J_{1}=5$ kg· m^2^, $J_{2}=5$ kg· m^2^ and g=9.81 m/s^2^. The perturbation term is set to $\tau_{d}={\left[-0.4\sin(t),0.3\sin(t)\right]}^{T}$. The parameters in the controller are $k_{s}=250$, $\alpha_{1}=3.2$, and $\alpha_{2}=5.75$. The reservoir dimension is 100, with input and reservoir weight matrix parameters set to ν=0.2 and ρ=0.96, respectively. The update law parameters for the output weights are $k_{n}=0.001$ and $k_{w}=1.5$. For intrinsic plasticity, the learning rate is η=0.0008 and the expected distribution mean is μ=0.4. The initial states are defined as $q\left(0\right)={\left[0.5,1.2\right]}^{T}$ and $\dot{q}\left(0\right)={\left[1.5,1.5\right]}^{T}$, while the desired trajectories are $q_{d1}=\sin\left(t\right)$ and $q_{d2}=\cos\left(t\right)$.

**Figure 3:**
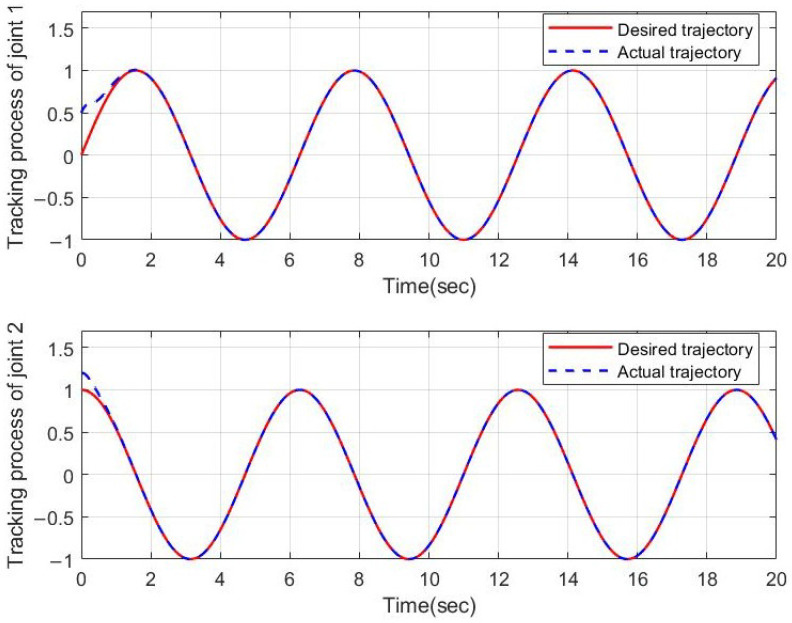
Tracking process under the proposed control scheme for Scenario 1.

**Figure 4:**
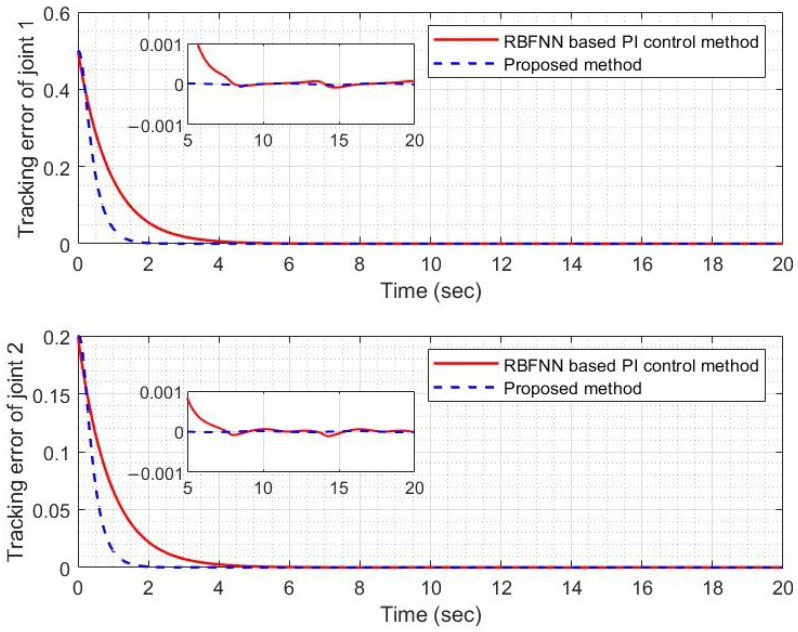
Tracking errors comparison between the RBFNN-based PI control method [24] and proposed method for Scenario 1.

**Figure 5:**
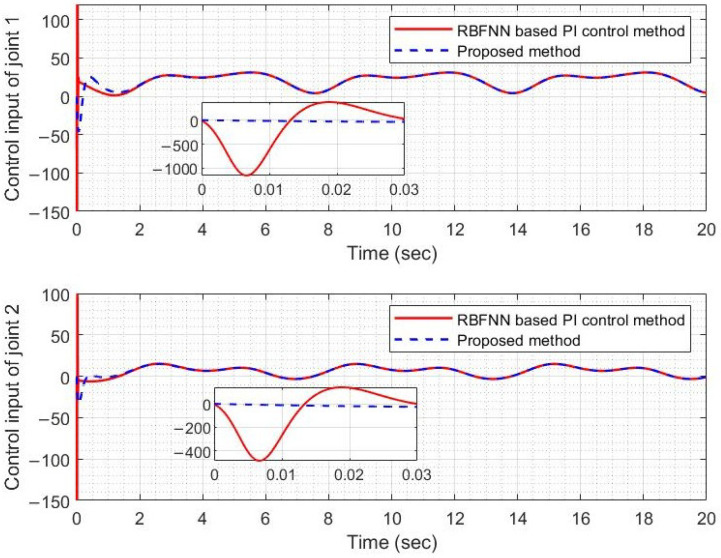
Control input comparison between the RBFNN-based PI control [24] method and the proposed method for Scenario 1.

**Figure 6:**
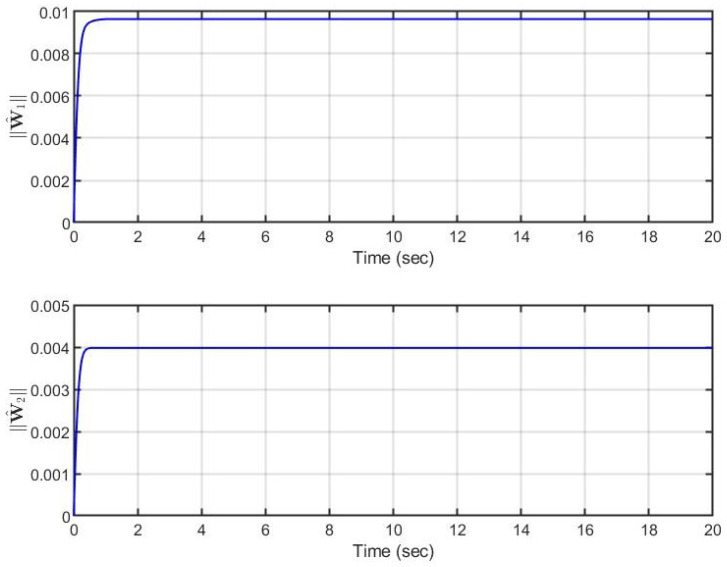
Norms of IP-driven cycle ESN output weight for Scenario 1.

The simulation results are presented in Figure 3, Figure 4, Figure 5 and Figure 6 and Table 2, Table 3 and Table 4. Specifically, Figure 3 illustrates the trajectory tracking performance, showing that the system effectively tracks the desired trajectory after a short transient period. Subsequently, Figure 4 and Figure 5 compare the control performance of the RBFNN-based PI control method [24] with that of the developed IP-driven cycle ESN under the same system model, identical reference trajectories, and identical initial conditions. Figure 4 illustrates the tracking errors of the two joints under different control methods. It can be seen that the proposed method exhibits a significantly faster convergence rate and achieves smaller transient tracking errors. The same result can also be observed from Table 2 and Table 3, where the tracking accuracy and convergence rate are quantitatively evaluated. Specifically, the integral of absolute error $\int \left|e_{i}\left(t\right)\right|dt$ (i=1,2) is employed to measure the tracking precision, while the integrated squared error $\int {\left(e_{i}\left(t\right)-{\bar{e}}_{i}\right)}^{2}dt$ serves as a metric for tracking smoothness, with ${\bar{e}}_{i}$ denoting the mean error. The quantitative results verify that the proposed method significantly enhances the steady-state accuracy while achieving a faster convergence rate across all joints. To visually illustrate the control behaviour, the trajectories of the control inputs are plotted in Figure 5. It can be seen that the proposed method generates significantly smoother control inputs than the RBFNN-based PI control method, particularly during the initial transient stage, where it effectively suppresses abrupt control variations and avoids large control peaks. After the transient response, both methods produce similar control signals to accomplish trajectory tracking. The quantitative comparisons presented in Table 4 further confirm these observations, demonstrating that the proposed method achieves smoother control inputs while maintaining comparable control effort. Figure 6 shows the evolution of the output weight norms of the proposed method. The norms of the output weights for both joints show initial transient adjustments and then converge to bounded, stable values, verifying that the adaptive learning rule ensures the stability of the network parameters during the control process.

- Simulation Scenario 2

In this scenario, the expressions for M(q), $C(q,\dot{q})$, G(q), $F(\dot{q})$ and $\tau_{d}$ are as follows [25]:

$$M\left(q\right)=\left[\begin{array}{cc} p_{1}+p_{2}+2p_{3}\cos\left(q_{2}\right) & p_{2}+p_{3}\cos\left(q_{2}\right)\\ p_{2}+p_{3}\cos\left(q_{2}\right) & p_{2} \end{array}\right]$$

$$C\left(q,\dot{q}\right)=\left[\begin{array}{cc} -p_{3}{\dot{q}}_{2}\sin\left(q_{2}\right) & -p_{3}\left({\dot{q}}_{1}+{\dot{q}}_{2}\right)\sin\left(q_{2}\right)\\ p_{3}{\dot{q}}_{1}\sin\left(q_{2}\right) & 0 \end{array}\right]$$

$$G\left(q\right)=\left[\begin{array}{c} p_{4}\cos\left(q_{1}\right)+p_{5}\cos\left(q_{1}+q_{2}\right)\\ p_{5}\cos\left(q_{1}+q_{2}\right) \end{array}\right]$$

$$F\left(\dot{q}\right)=0.2\sin\left(\dot{q}\right)$$

$$\tau_{d}=\left[0.2\sin(t);0.2\sin(t)\right]$$

$$p=\left[p_{1},p_{2},p_{3},p_{4},p_{5}\right]=\left[3.5,0.76,0.87,3.04,0.87\right].$$

The controller parameters are chosen as $k_{s}=50$, $\alpha_{1}=2.4$, and $\alpha_{2}=3.5$. The neural network parameters are set as follows: the dimension of the reservoir is 150, the positive constants in the input layer and reservoir weight matrices are ν=0.2 and ρ=0.96, the parameters of the neural network output weight update law are $k_{n}=0.1$ and $k_{w}=0.1$, the intrinsic plasticity learning rate is η=0.0008, and the expected distribution mean is μ=0.4. The initial states *q* and $\dot{q}$ of the system are $q\left(0\right)={\left[0.5,1.2\right]}^{T}$ and $\dot{q}\left(0\right)={\left[1,0\right]}^{T}$, while the desired trajectories are set as $q_{d1}=\sin\left(t\right)$ and $q_{d2}=\cos\left(t\right)$.

**Figure 7:**
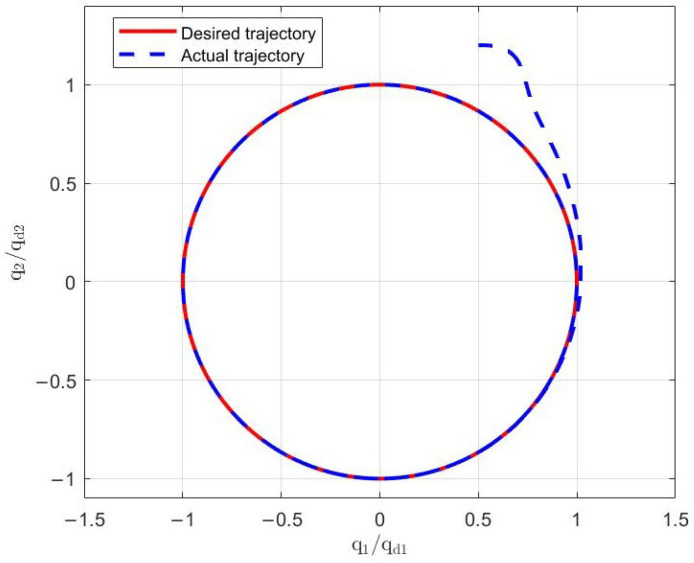
Tracking process under the proposed control scheme for Scenario 2.

**Figure 8:**
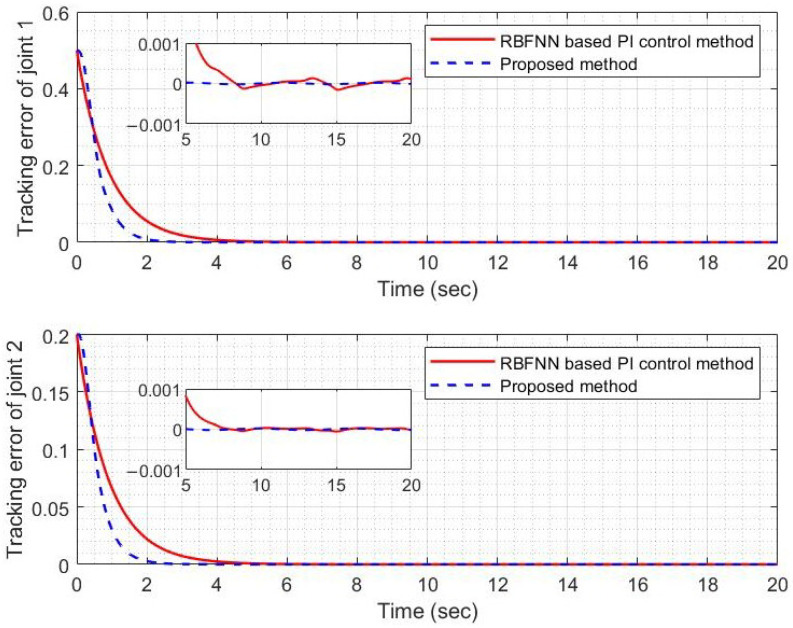
Tracking errors comparison between the RBFNN-based PI control method [24] and proposed method for Scenario 2.

**Figure 9:**
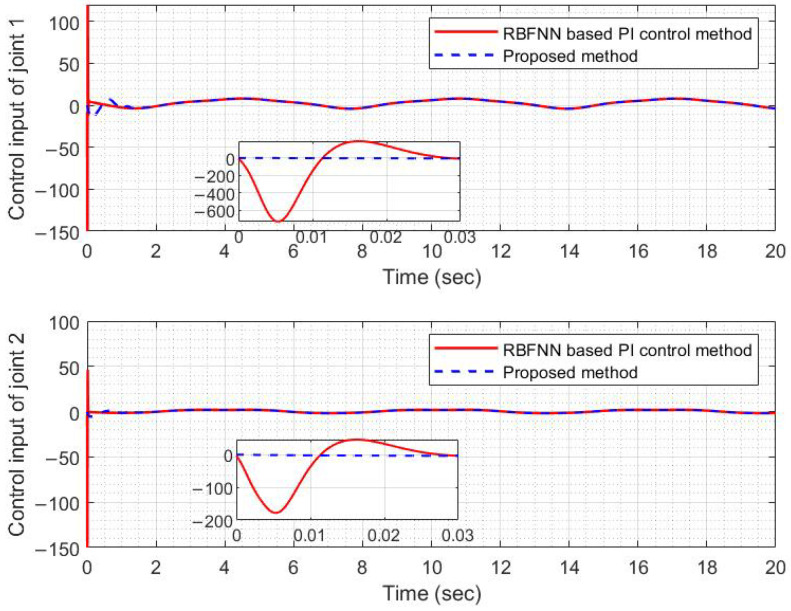
Comparison of the control inputs generated by the proposed method and RBFNN-based PI control method [24] for Scenario 2.

**Figure 10:**
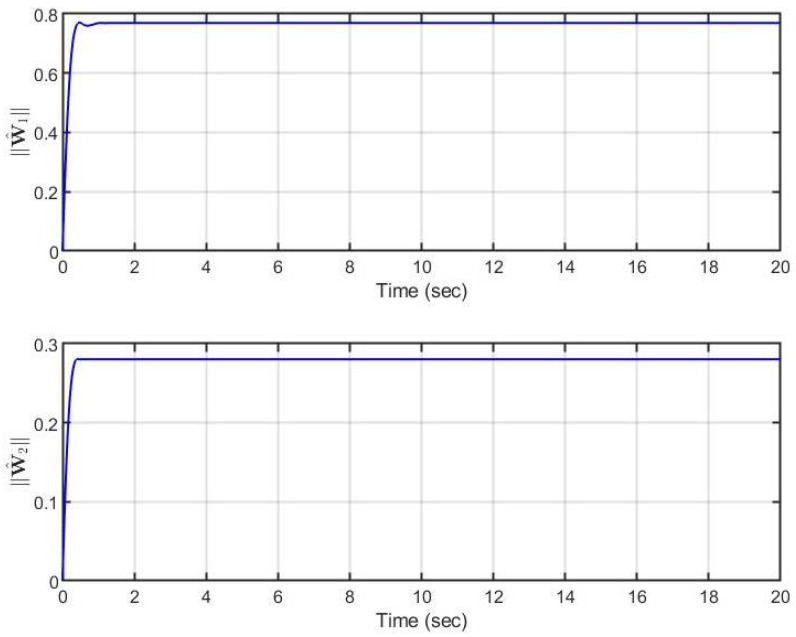
Norms of IP-driven cycle ESN output weight for Scenario 2.

The simulation results are illustrated in Figure 7, Figure 8, Figure 9 and Figure 10 and Table 5, Table 6 and Table 7. As depicted in Figure 7, the system tracks the desired trajectory effectively. Similarly, Figure 8 illustrates the tracking error responses of both joints. Compared with the RBFNN-based PI control method, the proposed method provides faster transient convergence and smaller steady-state error bounds. The enlarged views further confirm its tighter convergence toward zero. This enhancement in tracking precision is mathematically verified by the tracking error performance metrics in Table 5, which demonstrate lower integrated absolute errors and smaller variances for the proposed approach. Furthermore, Table 6 evaluates the system’s transient responsiveness. It shows that the proposed method achieves shorter settling times for both joints. Finally, to illustrate the corresponding control behavior, the trajectories of the control inputs are plotted in Figure 9. Although both methods achieve smooth steady-state control, the proposed method effectively reduces the initial control peaks, leading to a smoother transient response. This noticeable improvement in control smoothness is fully supported by the performance metrics presented in Table 7, where the reduced variance values confirm that the proposed scheme effectively suppresses undesired oscillations while maintaining a more economically efficient control energy expenditure. Figure 10 shows the evolution of the output weight norms of the IP-driven cycle ESN. The weight norms rapidly converge to constant values and remain bounded throughout the control process, demonstrating the stability of the proposed adaptive learning scheme.

## 5. Discussion

The simulation results presented in Section 4 demonstrate the superior performance of the proposed method compared with the RBFNN-based PI control approach in terms of tracking accuracy, convergence speed, and control input smoothness. As shown in Figure 3, Figure 4, Figure 7 and Figure 8 and quantified in Table 2 and Table 5, the proposed scheme achieves higher tracking precision. Moreover, the shorter settling times reported in Table 3 and Table 6 indicate a faster convergence rate. In addition, the smoothness of the generated control inputs is clearly demonstrated by Figure 5 and Figure 9 and quantitatively validated by Table 4 and Table 7.

Despite these advantages, several limitations of this study should be recognized. First, the theoretical validation and performance evaluations were established under ideal numerical simulation environments, which omit critical non-ideal practical constraints such as high-frequency sensor noise, actuator dead zones, and discrete sampling delays. Second, the reference trajectories tested in this work are entirely smooth continuous functions. In realistic industrial applications, controllers must frequently handle sudden trajectory modifications or non-smooth command abruptions. Addressing these practical gaps by enhancing the controller’s resilience to discontinuous trajectories and conducting physical validation on an experimental hardware platform will be the primary focus of our subsequent research.

## 6. Conclusions

This paper develops an IP-driven cycle ESN integrated with RISE robust control to realize asymptotic trajectory tracking of uncertain robotic manipulators, where the cycle reservoir cuts network computation burden and intrinsic plasticity improves the network’s approximation ability for time-varying nonlinearities, the adaptive law prevents neural weight drift and the RISE term compensates residual approximation errors and external disturbances, and Lyapunov analysis rigorously proves closed-loop signal boundedness and asymptotic convergence of tracking errors. Quantitative results from two simulation scenarios demonstrate that the proposed method outperforms the RBFNN-based PI control approach in terms of tracking accuracy, convergence speed, and control input smoothness. However, this study possesses several inherent limitations. First, all validation results were obtained under ideal numerical simulations without considering non-ideal factors such as practical sensor noise, actuator dead zones, and sampling delays. Second, the reference signals considered in this work are entirely smooth functions, without considering trajectories with some abrupt changes. To address these drawbacks, future work will be dedicated to improving our controller to adapt to abrupt trajectory changes and conducting physical hardware experiments.

## Figures and Tables

**Table 1:** Implementation steps of the proposed control scheme.

Step	Core Implementation Task
Step 1	Initialize robot initial states, controller gains and IP-cycle ESN hyperparameters.
Step 2	Measure joint position q(t) and velocity $\dot{q}\left(t\right)$; construct tracking error $e_{1}$, filtered error $e_{2}$ and auxiliary filtered error *r*.
Step 3	Build network input $z\left(t\right)={\left[q^{T}\left(t\right),{\dot{q}}^{T}\left(t\right),q_{d}^{T}\left(t\right),{\dot{q}}_{d}^{T}\left(t\right),{\ddot{q}}_{d}^{T}\left(t\right),e_{1}^{T}\left(t\right),e_{2}^{T}\left(t\right),{\dot{e}}_{1}^{T}\left(t\right)\right]}^{T}$, update reservoir neuron gain $a_{j}\left(t\right)$ and bias $b_{j}\left(t\right)$ via intrinsic plasticity online learning rules.
Step 4	Use IP-driven cycle ESN to approximate unknown lumped nonlinearity N(·), and update ESN output weight $\hat{W}$ by the adaptive law.
Step 5	Compute the composite control input *u*, and apply the torque to the manipulator.
Step 6	Repeat Step 2 to Step 5 in real time until the tracking error asymptotically converges to zero.

**Table 2:** Quantitative comparison of tracking performance indicators for Scenario 1.

	$\int \left|e_{1}\left(t\right)\right|\mathrm{dt}$	$\int \left|e_{2}\left(t\right)\right|\mathrm{dt}$	$\int {\left(e_{1}\left(t\right)-{\bar{e}}_{1}\right)}^{2}\mathrm{dt}$	$\int {\left(e_{2}\left(t\right)-{\bar{e}}_{2}\right)}^{2}\mathrm{dt}$
RBFNN-based PI control method [24]	0.4546	0.1822	0.1032	0.01652
Proposed method	0.2366	0.0947	0.0712	0.0117

**Table 3:** Comparison of settling times under different methods for Scenario 1.

	Settling Time of Joint 1	Settling Time of Joint 2
RBFNN-based PI control method [24]	5.6739	4.8318
Proposed method	2.1445	1.835

**Table 4:** Performance metrics for control signals under different methods for Scenario 1.

	$\int \left|u_{1}\left(t\right)\right|\mathrm{dt}$	$\int \left|u_{2}\left(t\right)\right|\mathrm{dt}$	$\int {\left(u_{1}\left(t\right)-{\bar{u}}_{1}\right)}^{2}\mathrm{dt}$	$\int {\left(u_{2}\left(t\right)-{\bar{u}}_{2}\right)}^{2}\mathrm{dt}$
RBFNN-based PI control method [24]	428.02	150.35	9717.4	2105.2
Proposed method	427.96	145.67	1913.9	799.37

**Table 5:** Quantitative comparison of tracking performance indicators for Scenario 2.

	$\int \left|e_{1}\left(t\right)\right|\mathrm{dt}$	$\int \left|e_{2}\left(t\right)\right|\mathrm{dt}$	$\int {\left(e_{1}\left(t\right)-{\bar{e}}_{1}\right)}^{2}\mathrm{dt}$	$\int {\left(e_{2}\left(t\right)-{\bar{e}}_{2}\right)}^{2}\mathrm{dt}$
RBFNN-based PI control method [24]	0.4552	0.1821	0.1033	0.0165
Proposed method	0.3236	0.1244	0.1004	0.0153

**Table 6:** Comparison of settling times under different methods for Scenario 2.

	Settling Time of Joint 1	Settling Time of Joint 2
RBFNN-based PI control method [24]	5.6974	4.8364
Proposed method	2.8468	2.4276

**Table 7:** Performance metrics for control signals under different methods for Scenario 2.

	$\int \left|u_{1}\left(t\right)\right|\mathrm{dt}$	$\int \left|u_{2}\left(t\right)\right|\mathrm{dt}$	$\int {\left(u_{1}\left(t\right)-{\bar{u}}_{1}\right)}^{2}\mathrm{dt}$	$\int {\left(u_{2}\left(t\right)-{\bar{u}}_{2}\right)}^{2}\mathrm{dt}$
RBFNN-based PI control method [24]	87.353	29.357	2947.2	210.78
Proposed method	84.162	28.495	345.13	48.375

## Data Availability

The simulation data generated and analyzed during the current study are available from the corresponding author upon reasonable request.
